# Adaptive Biological Neural Network Control and Virtual Realization for Engineering Manipulator

**DOI:** 10.1155/2022/2424279

**Published:** 2022-08-29

**Authors:** Hao Guo, Hongyang Liu, Dashuai Zhou, Yao He

**Affiliations:** ^1^Departmentof Mechanical Manufacturing, School of Mining and Mechanical Engineering, Liupanshui Normal University, Liupanshui City, Guizhou Province 553004, China; ^2^Department of Mining Engineering, School of Mining and Mechanical Engineering, Liupanshui Normal University, Liupanshui City, Guizhou Province 553004, China

## Abstract

By analyzing the feasibility of the digital twin technology in the assembly of construction machinery, the assembly process of the construction manipulator in the engineering environment is discussed. According to the application criteria and modeling requirements of digital twin, the overall framework of digital twin engineering manipulator assembly modeling and simulation is constructed from three aspects: model layer, data layer, and application layer. According to the operation task characteristics of space engineering manipulator, the feasibility of the control method based on joint angular velocity is analyzed, and the task environment of space engineering manipulator based on Markov model is defined. Aiming at the application of the algorithm in the control task of the space engineering manipulator, a reward function with the addition of the angular velocity soft bound term is designed, which improves the strategy optimization process of the algorithm and obtains a better control effect of the engineering manipulator. The motion trajectory of the end of the engineering manipulator is directly given on the simulation platform, and the expected motion of each joint of the engineering manipulator is calculated through the kinematics of the engineering manipulator. It can be seen from the simulation results that the controllers designed in this study can achieve ideal control effects. With the help of Baxter robot platform, the control algorithm designed in this study is applied to the actual engineering manipulator control, and the effectiveness of the control algorithm is further proved by the actual control effect.

## 1. Introduction

As the most commonly used robot in the industry, the construction manipulator is mostly controlled by point-to-point control in the early stage [1]. This control scheme is suitable for scenarios with low precision requirements. With the increase of production technology, industrial manufacturing puts forward higher requirements for the control accuracy of the construction manipulator, and at the same time requires the end of the construction manipulator to track the given reference trajectory motion [2]. On the other hand, traditional engineering manipulators are generally made of rigid materials with large volume and mass, which cannot complete high-precision work tasks, and are often used for mechanical work such as handling or assembly. With the advent of the era of intelligent manufacturing, the work tasks of the construction manipulator have become refined, and industrial production has put forward higher requirements for the precision of the construction manipulator [3]. At the same time, the adaptability of the production line to different work tasks is also increasing, and the scenarios in which humans and robots cooperate to complete production tasks are gradually increasing [4].

In order to improve the control accuracy and ensure the safety of human-computer interaction, a new type of engineering manipulator system, the flexible engineering manipulator system, is proposed. Flexible engineering manipulators are divided into flexible link engineering manipulators and flexible joint engineering manipulators [5]. The flexible link engineering manipulator is that the link of the engineering manipulator is composed of elastic materials, and the connecting rod itself has the characteristics of flexibility; the flexible joint engineering manipulator is that the connecting rod of the engineering manipulator is still composed of rigid materials but exists at the joints of the engineering manipulator. The spring device makes its joint flexible. This study will take the flexible joint engineering manipulator as the research object. Compared with the rigid construction manipulator system, the flexible joint construction manipulator has the advantages of light structure, high control precision, high load-to-weight ratio, and quick response. At the same time, due to the elastic brakes installed at the joints, it has good flexibility [6]. When encountering obstacles during the movement, its contact force will be much smaller than that of the rigid engineering robot arm, so it can effectively protect the operator [7].

To realize the precise control of the engineering manipulator, it is necessary to model the system accurately and then carry out state feedback control through modern control theory [8]. However, in practical applications, the kinematics and dynamic models of engineering manipulators inevitably have uncertainties, and it is difficult to achieve accurate modeling. For the problem of uncertain items in the system, there is usually a scheme to identify the unknown nonlinearity of the system by using fuzzy logic or neural network. However, it is a cumbersome process to train the neural network and adjust the parameters. At the same time, in actual production, it is very common for the engineering manipulator to perform the same or similar tasks repeatedly. How to save the knowledge of neural network identification system dynamics, realize the learning of system dynamics, and avoid repeated training of neural network is a research direction of great theoretical significance. Because of the limitations of sensor deployment and the influence of external interference, we often cannot obtain all the state variables of the system. Therefore, it is of great theoretical and practical value to study the controller design of the flexible joint engineering manipulator system whose model contains unknown dynamics and unobtainable state quantities.

This study expounds on the main problems existing in the design stage of the current engineering manipulator assembly process and analyzes the necessity of introducing digital twin technology into traditional assembly. By analyzing the application requirements of digital twin assembly, the main process of construction manipulator assembly under the background of digital twin is planned, and the construction of the construction manipulator assembly process based on digital twin is completed from the model layer, data layer, and application layer. This section introduces a deep deterministic policy gradient algorithm for continuous motion control in the control of space engineering manipulators for the multi-degree-of-freedom control system of space engineering manipulators. The method of adding the angular velocity soft bound to the reward function effectively solves the problem of neural network divergence. The simulation results show that the engineering manipulator can be stably controlled at the target point. The engineering manipulator controller designed in this study is tested on the engineering manipulator platform with two degrees of freedom as an example. Through the engineering manipulator platform, from the ideal system model and state to the unfavorable application conditions of the unknown system model and state, it is proved that the control algorithm proposed in this study can achieve the ideal control effect. Moreover, the adaptive control based on the RBF neural network can effectively fit the unknown model under the condition of unknown system model and achieve an ideal control effect. Finally, the Baxter robot arm system is used to verify the two adaptive neural network control algorithms designed in this study.

## 2. Related Work

For the control of engineering manipulators, a local linearization method is usually used to linearize the nonlinear part of the dynamics of the engineering manipulator near the target trajectory [9]. However, due to the strong time-varying, nonlinear, and strong coupling characteristics of engineering manipulators, local linearization cannot guarantee the global stability of the system. In order to solve this problem, some scholars have proposed a feedback linearization method [10]. This method mainly uses differential geometry and spatial coordinate transformation to make the input and state or input and output of the nonlinear system approximately satisfy the linear relationship and then use mature linearity. The system control method makes the system satisfy a certain robustness. Related scholars discussed the problem of robust tracking control of rigid engineering manipulators with uncertain dynamics using nominal feedback controllers and variable structural compensators [11]. The results show that the method can eliminate the influence of large system uncertainty and ensure the asymptotic convergence of the output tracking error. The researchers further, using a multiloop version of the small gain set, can obtain robust trajectory tracking under the assumption that the deviation of the model from the real system satisfies some norm inequalities [12]. Related scholars use gap metric analysis to derive the robustness and performance margins of feedback linearized controllers [13]. Unlike previous stability analyses, it incorporates the case of outputting nonstructural uncertainties and derives general stability conditions that can be applied to stable and unstable systems.

Related scholars use the specific structure of engineering manipulator dynamics to develop a simple global convergence adaptive controller, design PD feedback part and full dynamic feedforward compensation part, perform online estimation of unknown manipulator and payload parameters, this method is simple to calculate, the joint accelerations are not known, and there is no need to estimate the inverse of the inertia matrix [14]. The researchers designed a compensatory control rate and a nonlinear filter feedback term to obtain a globally progressively stable tracking effect [15]. Related scholars have proposed a robust control method for n-link engineering manipulators with uncertain upper bounds [16]. This method does not need to identify all the physical parameters of the engineering manipulator but only needs to estimate several parameters of the upper bound function. For multilink engineering manipulators, this method is much less computationally intensive. However, the discontinuous control amount caused by this method increases, and it is difficult to suppress disturbance. Further, related scholars have introduced an estimation rate of exponential changes such as random parameters and tracking error, which further enhances the robustness of the system [17].

Relevant scholars pointed out that the inversion control algorithm is a control algorithm for high-order complex nonlinear systems [18]. It combines the design of the controller with the selection of the Lyapunov function and divides the high-order system into several low-order subsystems in series according to the order of the system. The inversion control algorithm reverses step by step for each low-order subsystem in series and finally obtains the output of the overall controller.

The work of the restricted engineering manipulator is constrained by the environment, and its position, speed, and other states need to be restricted, which puts forward high requirements for the control design of the engineering manipulator. In recent years, a large number of scholars have devoted themselves to the control research of nonlinear systems with constraints and have achieved rich research results. At present, there are two common methods to solve the constraint problem, one is the obstacle Lyapunov function, and the other is the method based on function transformation [19].

The method based on function transformation adopts a class of nonlinear functions to directly transform restricted objects into equivalent unrestricted objects and then performs control design for the unrestricted objects. This method does not need to indirectly realize the constraint effect through the limited error but directly converts the limited physical quantity, so the control scheme is less conservative. Related scholars use the method based on function transformation to transform the tracking error of the system, so that the tracking error can reach the preset transient performance [20]. Subsequently, the method was further extended to solve the constrained tracking control problem of a class of strict feedback systems by combining different control techniques such as adaptive control, dynamic surface control, and neural network control.

## 3. Methods

### 3.1. Analysis of Engineering Manipulator Assembly Process Design Problems

In the field of construction machinery product assembly, with the gradual application of intelligent manufacturing theory and computer-aided process design, the digitization and visualization of product assembly process have higher requirements. As an important working device of large-scale construction machinery, the construction manipulator contains a variety of parts and connectors, with complex structure and various forms, and has extremely high requirements for design accuracy. The primary task of the assembly process design work is to meet the assembly quality of the product and then save the assembly cost and shorten the assembly cycle as much as possible through system planning and deployment. As the most intuitive quality evaluation index of assembly process documents, assembly accuracy is not only related to the manufacturing accuracy of parts but also affects the overall economy of the assembly system. At this stage, most manufacturing companies still have many problems in the formulation of assembly process regulations. For the field of engineering manipulator assembly, it is mainly reflected as follows:

1. In the assembly process of the engineering manipulator, the traditional two-dimensional assembly design method lacks the real-time acquisition and processing of dynamic data, the synchronization between the assembly process and the data update is poor, and there are also many shortcomings in theoretical methods. Accurate prediction and judgment of failure phenomena and failure causes are realized, and the system flexibility and real-time performance are poor.
2. The construction manipulator is assembled as a moving component. Compared with the conventional assembly, the mechanism assembly has the following characteristics:
3. ①Affected by the motion characteristics and force characteristics of the construction manipulator during operation, a certain deviation will accumulate between the components, so a certain assembly gap needs to be reserved at the assembly connection to avoid affecting the subsequent work stability. ②Due to the influence of the manufacturing accuracy of the parts during the matching process of the construction manipulator, there may be a certain deviation in the actual assembly state; that is, there is a matching gap. In addition, the parts that cooperate with each other will generate various deviations and change directions during the movement process, forming different deviation transmission paths and making the accuracy prediction of the mechanism more difficult.
4. There is a certain backwardness in the assembly of traditional engineering robotic arms in terms of assembly tools and assembly methods, resulting in an increase in the demand for manpower and material resources in the assembly process and poor economy. At the same time, the automation of assembly equipment is low, resulting in low production efficiency and insufficient production capacity.

### 3.2. Assembly Process of Construction Manipulator in Engineering Environment

With the rapid development of digital manufacturing technology, all kinds of intelligent equipment and virtual manufacturing technology are gradually applied to the field of traditional construction machinery assembly. The traditional manufacturing model has undergone a new transformation, forming a human-machine interactive collaborative analysis and decision-making integrated system. As an important new technical means in the field of intelligent manufacturing, digital twin is mainly used to realize the interconnection of physical space and digital space by relying on models, data, and sensors and digitally define and analyze the characteristics and activities of physical entities in virtual models. The digital twin model mainly consists of three parts, including digital space, physical space, and the correlation mechanism between the two. Combined with the actual assembly conditions of the production site and a large amount of data information in the physical manufacturing process, the dynamics is realized through the information interface between the two parts.

The role of the basic elements of physical space construction in digital twin assembly is to provide model and process support for the construction of digital space and provide mutual feedback with digital space to achieve program optimization. In the physical space, it is necessary to clarify the implementation steps of the assembly process and the tool requirements of the assembly site. Through the assembly sequence planning and assembly process planning of the construction robot arm, the actual operation is strictly carried out according to the assembly requirements, and other smart labels can be configured to facilitate the recording and follow-up of information in the digital space.

**Figure 1:**
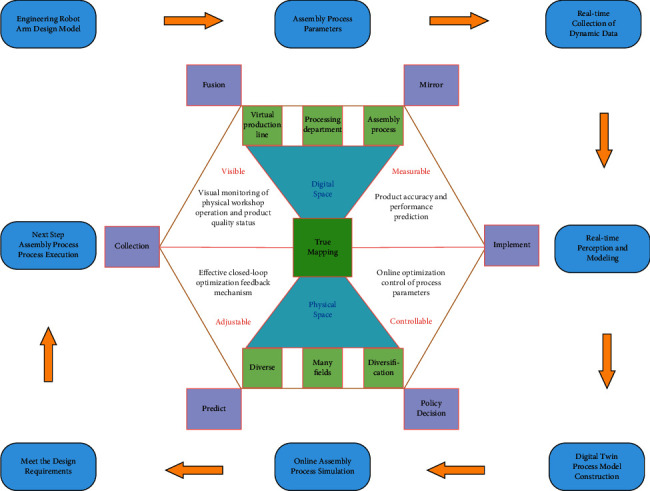
Digital twin assembly process control process diagram.

As a real mapping of physical space, digital space has roughly the same composition and structure as physical space. Virtual production lines, machining layouts, and assembly processes can be completed in third-party modeling and simulation software, including models of geometry, behavior, and rules, and related simulation, optimization, and analysis activities. The data information in the digital space is generally connected to the service platform through multiple external interfaces, forming a multifaceted and multidomain application of data integration and fusion.  Figure 1 shows the overall idea of digital twin assembly process control.

After reasonable collection and processing, the assembly process planning of the digital space is guided through the information interface. The engineering manipulator assembly based on digital twin mainly realizes the continuous iteration and optimization of assembly model design and assembly process parameters in the process of modeling and simulation.

Through dynamic data acquisition and modeling and simulation of the assembly process, the quality and accuracy of the model are affected. The assembly process parameters are analyzed, and continuous optimization feedback is carried out according to the condition update until the design requirements are met.

Through the research on the digital twin technology composition and digital twin assembly process, the digital twin-based engineering manipulator assembly process planning method is described in detail. In the process of engineering manipulator assembly simulation, digital twin technology is used to visualize the process flow of the assembly site, evaluate the pros and cons of the scheme, analyze whether it is reasonable, and realize the precise control of the whole process from the design of the assembly model of the engineering manipulator to the assembly production.

### 3.3. General Framework of Digital Twin Engineering Robotic Arm Assembly

**Figure 2:**
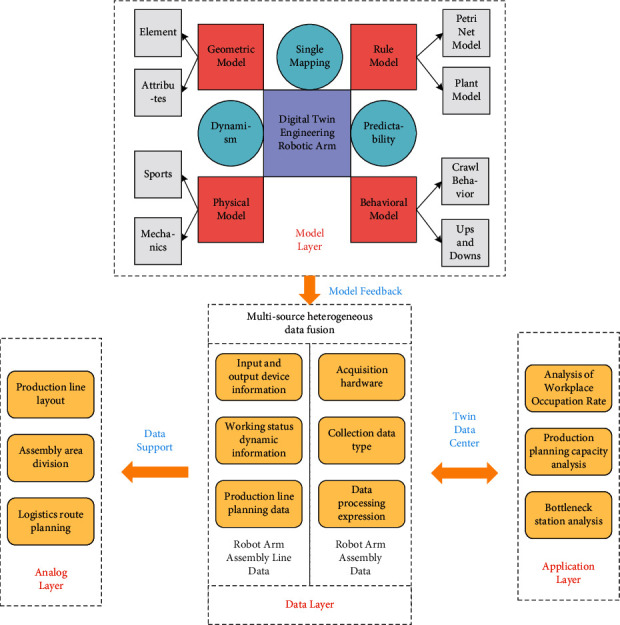
The overall framework of the digital twin engineering robotic arm assembly.

By summarizing the assembly process problems of the construction manipulator and the assembly process under the digital twin technology, combined with the preparation process of the assembly process of a certain type of construction manipulator, according to the assembly process design method, the assembly process design process of the construction manipulator in the engineering environment is based on its characteristics and characteristics. The composition is divided into three main parts:

1. *Data Layer*. We analyze the design size information, assembly process information, and dimensional tolerance information of the engineering robot arm and provide basic data support for the construction of the engineering robot arm assembly model and the control of the assembly process. Through data acquisition methods such as human-computer interaction, hardware acquisition terminals, and sensors, the assembly resources included in the assembly process of the construction robot arm are equipped with intelligent cores such as electronic labels and barcodes, and the data in the assembly production process of the construction robot arm are collected to realize the assembly information.
2. *Model Layer*. When facing the assembly process of the engineering manipulator, it is necessary to fully consider the multilevel information model in the engineering environment and the needs of the physical assembly site. Starting from the three-dimensional model of the engineering manipulator, according to its assembly characteristics and technical requirements, the assembly level and assembly sequence of the product are determined, so as to obtain an accurate assembly model. By acquiring detailed assembly knowledge such as products, tooling equipment, process knowledge, and logistics, 3D modeling of the assembly production line is performed based on the digital simulation platform to realize the mapping and connection from the physical space to the digital space from the engineering manipulator body to the entire production and assembly process.
3. *Application Layer.* The design, assembly, and application of the engineering robot arm are a complete life cycle, and an intelligent virtual environment is built in combination with digital twin technology. Through simulation, the specific working conditions of the assembly process are analyzed and fed back to the staff in real time, so as to realize the control and optimization of production capacity and production bottlenecks. The overall framework of the system is shown in Figure 2.

Because in the actual assembly process, the final assembly quality of the product will be affected by some unpredictable actual factors. In order to ensure the dynamic unity of the digital twin and the physical entity, it is necessary to build a digital twin model with all the elements of the physical entity space and realize the data and information exchange between the two. The constructed digital twin model meets the following technical requirements:

1. *Single Mapping*. The virtual entity structure in the digital twin space needs to be in one-to-one correspondence with the physical product, and the geometric feature information (such as shape, size, and tolerance) and manufacturing process information contained in the physical entity must be in accurate representation in the digital twin.
2. *Dynamics*. As an effective judgment model for physical entities, the product digital twin is required to reflect the current state of the system in real time during the entire process of production and assembly, so it is necessary to ensure the dynamic unity of the two.
3. *Predictability*. The process execution process of the whole life cycle of product manufacturing can be established by building a virtual simulation environment, the possible design defects and performance defects can be predicted through the detected real-time data, and the parameters can be adjusted in time.

On the basis of the above framework, they obtain manufacturing resource data such as equipment, products, processes, and logistics and use 3D design tools to establish a digital virtual assembly production line.

### 3.4. Adaptive Biological Neural Network Learning Method for Discrete Action Output

The Q-learning algorithm is a widely used reinforcement learning algorithm that can be used to optimize strategies in solving Markov decision processes. Taking action *a*_*t*_ for the current state *s*_*t*_ will not only affect the immediate reward *r*_*t*_ but also affect the reward obtained in the future and obtain the Q-value corresponding to the state *s*_*t*_ and the action at

(1) $$\begin{array}{c} Q\left(s_{t},a_{t}\right)=\frac{1+\overset{n}{\underset{i=1}{\sum }}\gamma^{i}}{r_{t}}+\frac{1+\overset{n}{\underset{i=1}{\sum }}\gamma^{i}}{r_{t+1}}+\frac{1+\overset{n}{\underset{i=1}{\sum }}\gamma^{i}}{r_{t+2}}+\cdot \cdot \cdot +\frac{1+\overset{n}{\underset{i=1}{\sum }}\gamma^{i}}{r_{t+i}}\text{.} \end{array}$$

Among them, the discount function indicates that the current action will weaken the series of rewards obtained in the future as the time step increases.

The state for time *t *+* *1 is obtained as follows:

(2) $$\begin{array}{c} Q\left(s_{t+1},a_{t+1}\right)=\frac{1+\overset{n}{\underset{i=1}{\sum }}\gamma^{i}}{r_{t+1}}+\frac{1+\overset{n}{\underset{i=1}{\sum }}\gamma^{i}}{r_{t+2}}+\frac{1+\overset{n}{\underset{i=1}{\sum }}\gamma^{i}}{r_{t+3}}+\cdot \cdot \cdot +\frac{1+\overset{n}{\underset{i=1}{\sum }}\gamma^{i}}{r_{t+1+i}}\text{.} \end{array}$$

The Bellman iteration equation for the state-action value function can be obtained as follows:

(3) $$\begin{array}{c} Q\left(s_{t},a_{t}\right)=Q\left(s_{t+1},a_{t+1}\right)-\frac{1+\overset{n}{\underset{i=1}{\sum }}\gamma^{i}}{r_{t}}+\frac{1+\overset{n}{\underset{i=1}{\sum }}\gamma^{i}}{r_{t+1+i}}\text{.} \end{array}$$

In the Q-learning algorithm, the corresponding update equation is as follows:

(4) $$\begin{array}{c} \frac{Q\left(s_{t},a_{t}\right)}{\alpha_{t}\left(s_{t},a_{t}\right)}r_{t}\sqrt{\frac{\gamma \max_{a}Q\left(s_{t+1},a_{t+1}\right)}{Q\left(s_{t},a_{t}\right)}}⟶Q_{t+1}\left(s_{t},a_{t}\right)\text{.} \end{array}$$

The Q-table implements a mapping strategy from the state *s* to the optimal action. When the number of states of the environment is large, the storage space required by the Q table will become large. Neural networks can fit large nonlinear functions and have generalization capabilities. Instead of bulky Q tables, neural networks can be used to fit mapping functions from states to actions.

Taking the space engineering manipulator with 6 degrees of freedom as an example, if the motion of each joint is simply discrete into 3 actions of “forward rotation, reverse rotation, and stop,” the combination of actions generated by all 6 joints is 36 = 729. If a finer control is to be obtained, a correspondingly finer discrete action is required, and the number of actions generated will be greater. As a result, the adaptive biological neural network cannot be directly applied to the continuous motion control of the space engineering manipulator.

Deterministic strategy neural network can express deterministic control strategy, the input layer of neural network is state *s*, and the output is deterministic optimal action.

The deterministic policy gradient neural network does not need to calculate the action value corresponding to each action, so it does not need to discretize the action, so it can output the continuous control action of the multi-degree-of-freedom spatial engineering manipulator.

The deterministic policy gradient neural network adjusts the parameters of the neural network according to the policy optimization gradient. However, it is difficult to obtain the gradient direction of the policy optimization parameter update, which makes the training of the deterministic policy gradient neural network difficult to achieve.

### 3.5. Deep Deterministic Policy Gradient Algorithm Design

This study will use the deep deterministic policy gradient algorithm to learn and optimize the control strategy of the space robot arm. The DDPG algorithm is based on the actor-critic training system and can be adapted to the continuous control of multi-degree-of-freedom spatial engineering manipulators. The actor part uses a deterministic policy gradient neural network to output the continuous control action of the multi-degree-of-freedom engineering manipulator. The critic part uses an adaptive biological neural network to fit the action Q-value function.

The process of training the critical neural network is as follows: the critical action value function network is used to fit the action value function. The input of the neural network is the state *s*_*t*_ at time *t* and the action *a*_*t*_, and the output is the fitting value of the neural network. The target value uses the Bellman iteration equation Q. Therefore, the sum of squares of the difference between the target value and the fitted value is the loss value. The process of fitting the action value function by the network is to update the parameter C of the network to reduce the loss value *L*.

By adding a soft bound on the angular velocity to the reward function, the reward function is of the following form:

(5) $$\begin{array}{c} r=\frac{d}{\overset{n}{\underset{i=1}{\sum }}\max\;\left(0,v_{b}/\left|v_{i}\right|\right)}\text{.} \end{array}$$

## 4. Results and Analysis

### 4.1. Control Algorithm Simulation Platform Verification

The control system continuously obtains the parameters of the controlled object, the state of the system, the tracking error, and other information, compares it with the expected state and performance, and obtains the estimated value of the uncertain parameter according to the preset estimation rate. The estimated value is used as part of the controller, and the controller is then corrected so that the system achieves the desired tracking performance.

In this section, the controller will be simulated and verified on the engineering manipulator simulation platform. The derivative term of the expected trajectory involved in the simulation is described by the finite difference method. The specific form of the finite difference method to describe the derivative of a function is as follows:

(6) $$\begin{array}{c} \dot{f}\left(x_{i}\right)=\lim_{\Delta x⟶0}\frac{f\left(x_{i}\right)+f\left(\Delta x-x_{i}\right)}{\Delta x}\\ =\lim_{\Delta x⟶0}\frac{f\left(x_{i}+\Delta x\right)-f\left(x_{i}\right)}{\Delta x}\text{,}\\ \ddot{f}\left(x_{i}\right)=\lim_{\Delta x⟶0}\frac{f^{2}\left(x_{i}+\Delta x\right)-2f\left(x_{i}+\Delta x\right)f\left(x_{i}\right)+f^{2}\left(x_{i}\right)}{\Delta x^{2}}\text{.} \end{array}$$

**Figure 3:**
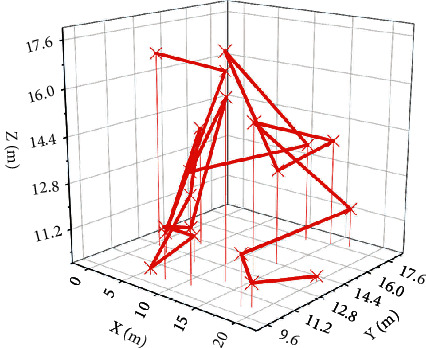
3D diagram of the motion of the construction manipulator.

First, the control effect of the controller based on the system model is tested. The motion effect diagram of the engineering manipulator is shown in Figure 3. It can be seen from these figures that the model-based controller can effectively control the motion of the engineering manipulator and can make its end track the given trajectory well, especially from the detailed diagram of the trajectory tracking of the end of the motion of the engineering manipulator. The actual motion trajectory of the end of the engineering manipulator is almost completely fitted with the expected trajectory.

The controller can control the engineering manipulator to move on the YOZ plane and make its execution end track the given desired motion trajectory. The trajectory of the end of the construction manipulator is tracked in detail, the actual trajectory of the end of the construction manipulator is not completely fitted with the expected trajectory, and there is a slight error. The error may come from the estimation error of the actual system model by the neural network and the choice of control parameters. In general, the controller can also achieve good control results when the system model is unknown.

The controller can control the construction manipulator to move on the YOZ plane and make the end of the construction manipulator track the given desired trajectory. The actual trajectory of the end of the engineering manipulator is not completely fitted with the expected trajectory, and there is a slight error. The error may originate from the estimation error of the actual system model by the neural network, the estimation error of the system velocity term by the state observer, and the selection of control parameters. But in general, when the system model is unknown and the system state quantity is unmeasurable, the controller can achieve a good control effect.

The control adjusts the network parameters in real time during the control process, and the parameters are kept within a certain range.

From the above test results of the three controllers, it can be seen that under ideal conditions, the model-based controller can achieve the best control effect, followed by the control effect of the adaptive neural network controller when the model is unknown. Finally, when the system model is unknown and the state quantity is unmeasurable, the control effect of the adaptive neural network controller with the state observer is adopted. In practical applications, if the system model and state are known, the model-based controller can be applied. However, in the actual system, the model is often not accurately determined, and the system state is not necessarily completely measurable, so the above two adaptive controllers have more practical application significance.

### 4.2. Verification of Control Algorithm Virtual Experiment Platform

In order to further illustrate and verify the effectiveness of the engineering manipulator control algorithm designed in this study, the designed controller is applied to the actual engineering manipulator system. Due to the complexity of the construction of the actual arm system and the progress of the project, the arm system of SRU3 has not been built yet and cannot be used for experimental verification of the control algorithm. Therefore, this study will borrow the existing Baxter robot as the experimental verification platform for the control algorithm.

The Baxter robot is designed with good safety and flexibility, which can facilitate application development and research, and can be flexibly used to deploy industrial production lines and scientific research in universities. The Baxter robot is a robot with two multi-degree-of-freedom structural arms. Each arm is a redundant structure with seven degrees of freedom, and its multiple degrees of freedom greatly improve the flexibility of the arm, which can better simulate the structure of the human arm, and can complete complex operation tasks.

For the convenience of description, the degrees of freedom of each joint of the arm are named. The joints of the arm are driven by a series of elastic drivers, which are different from the direct drive joints. This design can make the engineering manipulator have a certain flexibility and sensing ability. In the process of interacting with people, it can better ensure the safety of personnel. Among the seven joint degrees of freedom, the four joint degrees of freedom, S0, S1, S2, and E0, are mainly used to adjust the trajectory of the end of the arm movement. The three joint degrees of freedom of W0, W1, and W2 are mainly used to adjust the arm after reaching the desired trajectory. Compared with the designed SRU3 engineering manipulator system, the three degrees of freedom of S0, S1, and S2 can be compared to the three degrees of freedom of the shoulder joint of the SRU3 engineering manipulator. SRU3 is the two degrees of freedom of the wrist joint, and W3 is the redundant degree of freedom. S1 and E0 have the same axis of rotation, and the combined motion of the two joints is on the same plane, which makes it easier to see the motion effect.

The control system as a whole is divided into two parts, the development workstation and the robot main body. The development workstation is the user's computer, which is used to write and solve the robot control algorithm; there is an independent computer on the robot body, which is used to schedule robot tasks and generate control commands for the embedded controller on the robot. The mechanism executes the control instructions given by the user. The development workstation and the main robot computer are connected through Ethernet to control the robot.

Baxter is a robot platform developed based on the robot operating system. ROS is an open source system software with certain generality. It relies on the Linux operating system to manage computer resources and provides functions such as underlying driver management, task management and scheduling, and process communication scheduling for robots in the process of robot development. Based on the ROS system, it is convenient to use its interface for the secondary development of the robot. The computer system environment on the Baxter robot is “Gentoo + ROS + SDK,” which runs the ROS master independently to manage the robot's control tasks, robot status, and underlying hardware drivers and provides communication and development interfaces. Therefore, any computer capable of running ROS can connect to Baxter through the ROS network interface to operate and develop it. Baxter's SDK provides users with an interface to access underlying hardware such as robot motors and sensors through ROS. The computer system environment of the development workstation is “Ubuntu14.04 + ROS + SDK,” and the control algorithm of the robot can be developed and implemented on the user's computer.

**Figure 4:**
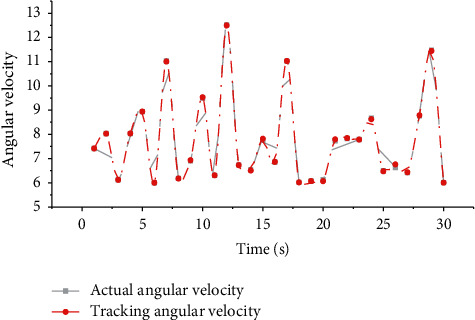
Angular velocity tracking renderings.

The joint trajectory tracking angular velocity of the action process of the model-based controller is shown in Figure 4. From the tracking effect diagram of the system output tracking the preset trajectory, it can be seen that the model-based controller can almost perfectly track the desired trajectory, and the tracking error is in the range of 0.01. The control input of the system is bounded and stable in the neighborhood of a certain value.

### 4.3. Experimental Results and Analysis

In this experiment, the S1 and E0 joints of the Baxter robot arm were selected as the controlled objects to verify the effectiveness of the controller. Considering the actual application conditions, it is impossible to establish an accurate system model. In this experiment, only the adaptive neural network controller based on state observation will be experimentally verified. Since the adaptive neural network controller does not need an exact system model or precise system parameters in the application process, it is possible to test the effect of the controller by directly inputting the desired trajectory for the joint without paying attention to the system parameters.

**Figure 5:**
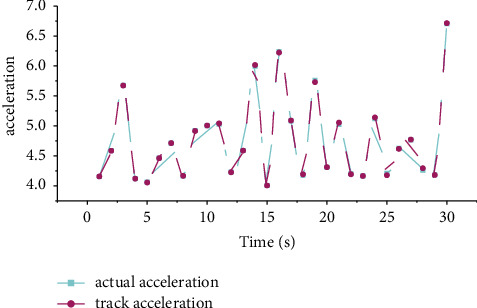
Adaptive neural network controls acceleration tracking effect.

**Figure 6:**
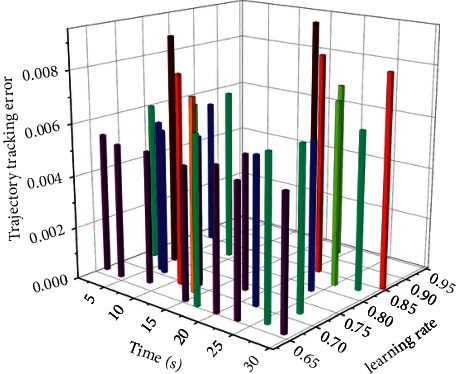
Adaptive neural network control trajectory tracking error.

Figure 5 shows the acceleration tracking effect of adaptive neural network control. It can be seen from the figure that the adaptive neural network controller designed in this study can control the engineering manipulator to track the trajectory after setting the appropriate control parameters. The actual tracking error is shown in Figure 6. It can be seen from the figure that the tracking error range is kept within 0.01.

**Figure 7:**
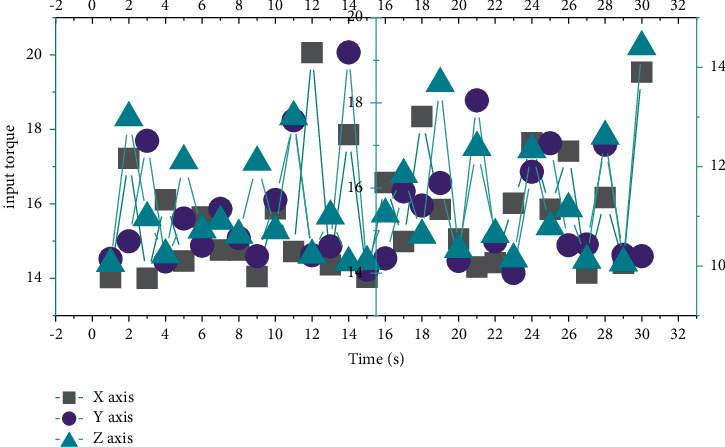
Adaptive neural network controls input torque.

Figure 7 shows the output torque of the controller. It can be seen that the adaptive neural network controller designed in this study can achieve a better control effect in the actual engineering manipulator system control.

**Figure 8:**
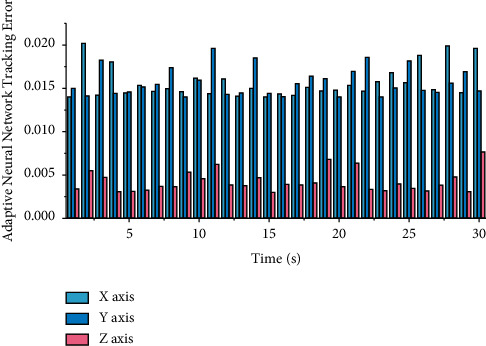
Adaptive neural network control trajectory tracking error based on state observation.

When the system state model is unknown and the system state is unmeasurable, appropriate control parameters are selected. The adaptive neural network controller based on state observation designed in this study can also control the engineering manipulator for trajectory tracking. The actual tracking error situation is shown in Figure 8. The absolute value of the maximum tracking error does not exceed 0.02/rad, and the conversion to angle value is also around 1.

The adaptive neural network controller designed in this study can also realize the desired trajectory tracking control of the engineering manipulator in the actual engineering manipulator system control.

Since too much information about the precise system model is not required, the above controller has good applicability and can be used to solve control problems of various similar engineering manipulator systems.

Comparing the tracking errors of the two controllers, it can be seen that the controller has a better control effect when the system state is measurable.

But at the same time, when the system model is unknown and the system state is unmeasurable, the adaptive neural network control strategy based on state observation can also provide an effective control scheme.

The above experimental results show that under the action of the two controllers, the trajectory tracking of the engineering manipulator has a certain error. The source of the error may have the following reasons: the controller has many parameters, which may not guarantee that the adjusted control parameters are optimal; at the same time, Baxter's arm joints are all flexible joints, and there will be certain changes in the movement process. The flexible vibration may also have a certain impact on the control accuracy.

## 5. Conclusion

In this study, a modeling and simulation framework of engineering manipulator assembly based on digital twin is designed. On the basis of summarizing the research status of digital twin and construction machinery assembly, the shortcomings of traditional construction machinery assembly are analyzed. According to the core concept, application criteria, and model requirements of digital twin, a design process and method of engineering manipulator assembly process based on digital twin are proposed. By studying the modeling requirements of the model layer, data layer, and application layer, the general idea of the digital twin assembly process management and control is put forward, and the assembly modeling and simulation framework of the engineering robot arm in the engineering environment is established. The kinematic control framework of the space engineering manipulator is designed, and the control input of the space engineering manipulator is the desired angular velocity of the joint. Since the control at the kinematic level is not affected by the dynamic parameters of the engineering manipulator, the dynamic parameter difference between the simulation platform and the real physical system has no effect on the transfer of the control strategy. Therefore, the simulation platform can be used to control a large number of engineering manipulators and collect data samples, and these data samples are used to train the deep policy neural network; after the neural network is trained, it can be directly transferred to the control of the real engineering manipulator system. It can achieve the same control effect as the simulation platform, thus avoiding the equipment wear and tear problem caused by relying on the real physical system to generate training data. This study designs a model-based control algorithm and an adaptive control algorithm for the engineering manipulator, considering the control requirements when the system model is known and the model is unknown. The adaptive method in the adaptive control adopts the radial basis function neural network and uses the fitting performance of the neural network to fit the engineering manipulator system. At the same time, the two situations of completely known and unknown system state are considered separately to solve the control under various environmental conditions in practical applications. The Lyapunov stability principle and MATLAB simulation are used to analyze and verify the effectiveness of the controller and system stability. Finally, before the construction of the engineering manipulator platform has been completed, the two adaptive neural network controllers designed in this study are experimentally verified with the help of the Baxter robot arm system, which provides further proof of the effectiveness of the algorithm.

## Data Availability

The data used to support the findings of this study are available from the corresponding author upon request.
